# Psoriasis and Connective Tissue Diseases

**DOI:** 10.3390/ijms21165803

**Published:** 2020-08-13

**Authors:** Toshiyuki Yamamoto

**Affiliations:** Department of Dermatology, Fukushima Medical and Dental University, Hikarigaoka 1, Fukushima 960-1295, Japan; toyamade@fmu.ac.jp

**Keywords:** psoriatic disease, innate immunity, SLE, dermatomyositis, systemic sclerosis, Sjögren syndrome, Still’s disease, sarcoidosis

## Abstract

Psoriasis is a chronic systemic inflammatory disease with various co-morbidities, having been recently considered as a comprehensive disease named psoriatic disease or psoriatic syndrome. Autoimmune diseases are one form of its co-morbidities. In addition to the genetic background, shared pathogenesis including innate immunity, neutrophil extracellular trap (NETs), and type I interferon, as well as acquitted immunity such as T helper-17 (Th17) related cytokines are speculated to play a significant role in both psoriasis and connective tissue diseases. On the other hand, there are definite differences between psoriasis and connective tissue diseases, such as their pathomechanisms and response to drugs. Therefore, we cannot expect that one stone kills two birds, and thus caution is necessary when considering whether the administered drug for one disease is effective or not for another disease. In this review, several connective tissue diseases and related diseases are discussed from the viewpoint of their coexistence with psoriasis.

## 1. Introduction

**Figure 1:**
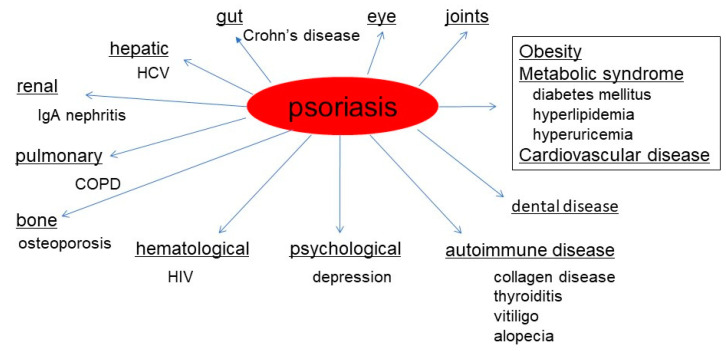
Psoriatic diseases showing various comorbidities.

Psoriasis is a chronic systemic inflammatory disease affecting not only the skin but also various internal organs. Recently, multisystemic involvements other than the skin and joints, such as gut, eye, and metabolic and cardiovascular systems, have been demonstrated in association with psoriasis (Figure 1). Psoriasis may not only be a skin-directed disorder, but also associated with systemic inflammatory features. Therefore, terms such as psoriatic disease or psoriatic syndrome have been proposed [1,2]. A similar concept is proposed as psoriatic march, laying stress on the time course of disease progression [3].

In particular, autoimmune disorders with significantly higher frequencies included rheumatoid arthritis (RA), alopecia areata, celiac disease, systemic sclerosis (SSc), Crohn’s disease, Sjögren syndrome (SjS), vitiligo, ulcerative colitis, systemic lupus erythematosus (SLE), and giant cell arteritis [4]. Common underlying immunological defects may be important in the pathogenesis of these complications. Autoimmune bullous disorders have also been reported, including bullous pemphigoid, anti-laminin gamma-1 (p-200) pemphigoid, and others. Additionally, SSc, SjS, sarcoidosis, autoimmune thyroiditis, alopecia, and vitiligo have also been associated.

Psoriasis is triggered by some external factors, such as mechanical stimuli (isomorphic response of Köbner), followed by a complex of self-DNA/RNA and cathelicidin(LL37) incorporated into plasmacytoid dendritic cells (pDCs), which then upregulates toll-like receptor-7 (TLR-7) and -9, leading to production of large amounts of interferon (IFN)-α. Interleukin 23 (IL-23), mainly produced by myeloid dendritic cells (mDC), plays a significant role in differentiating, amplifying, and maintaining Th17 differentiation of naïve T-cells. Additionally, IL-17 is produced by not only T-cells but also various other cells, such as innate cells. Thus, the IL-23/Th17 axis is the main stream of the inflammatory pathway of psoriasis. IL-17, IL-22, and IL-23 have been reported to play an important role not only in psoriasis but also in other autoinflammatory disorders.

The overlapping of psoriasis and connective tissue disorders has been occasionally reported [5]. In the current review, connective tissue diseases including SLE, SSc, dermatomyositis (DM), and SjS, as well as adult-onset Still’s disease, Behçet disease, and sarcoidosis, are discussed in terms of their relationship with psoriasis.

## 2. Psoriasis and SLE

Among the connective tissue diseases, SLE has often been reported in association with psoriasis [6,7,8,9,10]. The prevalence of SLE in patients with psoriasis has been reported to be estimated at 0.69% [9]. In a recent report from a lupus clinic in a single-center study, 63 psoriasis patients were observed among the 1823 SLE patients (3.46%). The 63 patients consisted of 49 females and 14 males (female/male = 3.5:1). Psoriasis was diagnosed at a mean of 9 years after the diagnosis of SLE, whereas psoriasis preceding SLE diagnosis was observed in only one case [10]. The types of psoriasis were plaque-type (87.3%), pustular type (4.8%), scalp psoriasis (7.9%), and psoriatic arthritis (PsA) (1.6%), suggesting that co-existence of SLE and PsA is rare. Childhood cases of psoriasis and SLE coexistence are extremely rare [11].

Several similarities have been suggested between psoriasis and SLE, including genetic, epigenetic, and pathogenic factors. The shared pathomechanisms include innate immunity, type I interferon, plasmacytoid dendritic cells, neutrophil extracellular trap (NETs), and Th1/Th17-type cytokines [12,13], which are supposed to play a significant role in the induction of both diseases. The co-existence of psoriasis and SLE has sometimes been observed, and both diseases share some common pathogenesis, such as Th1/Th17 type-dominant cytokine imbalance, pDC activation via TLRs, and IFN-α release. Type I interferon is known to drive cytotoxic cellular inflammation, and IFN-α induces expression of cutaneous lymphocyte antigen (CLA) on cytotoxic T-cells, helping their homing to the skin. CD123-positive pDC is observed in the lesional skin of LE [14,15]. As compared with SLE, association of cutaneous LE, such as LE profundus, has been rarely reported in association with psoriasis [16]. One of other similarities is comorbidities. Similar to psoriasis, SLE has also recently been suggested to be highly related to various comorbidities such as cardiovascular disease and metabolic syndrome [17]. Among connective tissue diseases, patients with SLE have impaired endothelial cells and compromised repair of the damaged endothelial cells [18], which may promote endothelial dysfunction and development of cardiovascular disease, as well as dysregulation of the innate immune response.

Recent studies of the pathogenesis of psoriasis have indicated that, following external triggers, a complex of self-DNA/RNA and LL37 is incorporated into plasmacytoid dendritic cells, which then upregulates IFN-α, leading to the induction of psoriasis. Making use of its mode of action in activating TLR7, imiquimod-induced psoriasis is frequently used in a mouse model for psoriasis. Although the morphology of topical imiquimod-induced lesional skin mimics human psoriasis, histopathology does not exhibit any of the aspects of human psoriasis. Furthermore, lesions induced by topical imiquimod treatment are transient, and are therefore different from human chronic psoriasis. In humans, several cases of imiquimod-induced de novo psoriasis or psoriasis-like lesions and exacerbation of pre-existing psoriasis have been reported to date [19]. In addition, systemic administration of imiquimod was reported to induce lupus-like symptoms in mice [20]. LL37, an endogenous antimicrobial peptide, has recently been suggested to be involved in SLE, as well as psoriasis. LL37 triggers IFN-α production in pDCs, and SLE patients had circulating T-cells responding to LL37, which correlated with anti-LL37 antibodies and disease activity [21]. As compared with psoriasis, LL37-specific T-cells in SLE displayed a T-follicular helper-like phenotype, implicating a pathogenic role in SLE [21].

By contrast, tumor necrosis factor-α (TNF-α) and IFN-α mutually exert inhibitory effects on each other, and biologics targeting TNF-α may be one of the possible candidates to modulate the immune balance via activation of nascent autoreactive T-cells, altered autoimmunity, imbalance between TNF-α and IFN-α, and induction of IL-17- and IL-22-producing CD4+ T-cells in the peripheral blood [22].

Alternatively, in a smaller number of cases, drugs can be attributed to the induction of other diseases such as (i) psoriasis induced by drugs used for SLE, or (ii) SLE or lupus-like lesions induced by biologics used for psoriasis (paradoxical reaction). Regarding therapies, caution is required because some drugs for one disease can sometimes deteriorate the other. Ultraviolet irradiation is effective for psoriasis, whereas it can worsen or trigger malar rash in SLE. Hydroxychloroquine is one of the standard drugs for SLE, which exerts its effects by suppression of TLRs and inhibition of type I cytokine production such as IFN-γ. By contrast, hydroxychloroquine sometimes deteriorates psoriasis [23]. Therefore, caution is necessary when we choose therapy in patients with both diseases. Regarding therapies using biologics, ustekinumab, which targets IL-12/23, is expected to have favorable effects on SLE [24], as well as psoriasis.

## 3. Psoriasis and SSc

**Figure 2:**
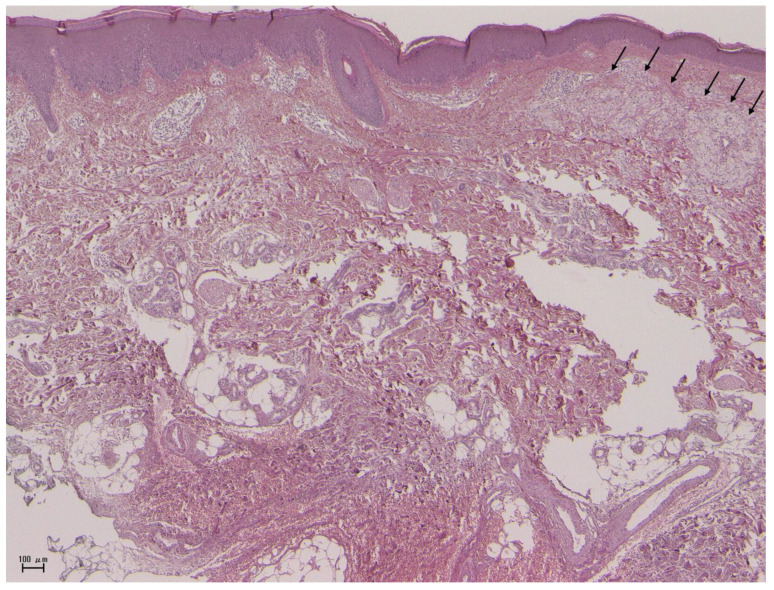
Biopsy specimen taken from a patient with psoriasis and SSc showing psoriatic epidermis (left side), dermal sclerosis, and xanthomatous foamy cells in the upper dermis (arrows). Scale bar: 100 μm.

Co-existence of psoriasis and SSc is rare, since previous studies have shown that psoriasis is Th1-dominant while SSc is a Th2-dominant disease [25]. In addition, histopathological collision of psoriasis and scleroderma has been observed relatively rarely (Figure 2). However, the recent growing body of evidence has shown that psoriasis is a Th1/Th17 disease, and that Th17 is also involved in SSc [26]. On the other hand, localized scleroderma in association with psoriasis is rare [27].

IL-17 subfamilies include IL-17A, IL-17B, IL-17C, IL-17D, and IL-17F. IL-17A is mainly involved in a number of autoimmune disorders. The serum levels of IL-17A and mRNA levels in peripheral blood of SSc patients are elevated [26]; however, the effects of IL-17 on fibrosis are controversial. In vitro, IL-17 stimulates fibroblast proliferation in SSc fibroblasts [26]. IL-17 has been reported to show no effects on collagen synthesis in SSc fibroblasts [26], while another study showed that IL-17A suppressed type I collagen expression [28]. IL-17A did not affect the induction of myofibroblasts [29]. In vivo studies have shown that IL-17A-deficient mice were partially protected by bleomycin-induced scleroderma [30], and bleomycin-induced scleroderma was attenuated by anti-IL-17A antibody [31]. These results suggest that IL-17 is one of the main targets for treating SSc [32,33,34]. By contrast, a recent study has shown that IL-17 softens the skin through induction of matrix metalloproteinase-1(MMP-1) [35]. These results may support the findings that psoriasis, in which IL-17A is increased, and SSc are rarely associated.

Innate immunity is important in both psoriasis and SSc. In psoriasis, IL-17 is secreted by various innate cells, such as γδT cells, neutrophils, mast cells, and NK T-cells. In SSc, mast cells increase in number in the scleroderma skin. Upon activation, degranulated mast cells produce a number of mediators such as inflammatory and fibrogenic cytokines. Innate lymphoid cell type 3 has been suggested to play a role in psoriasis and PsA [36], whereas type 2 is important in fibrosis [37]. Damage-associated molecular patterns (DAMPs)/alarmins are endogenous molecules released from necrotic or stressed cells to trigger subsequent immune responses. Various molecules such as high mobility group box 1 (HMGB-1), S100A8, S100A9, S100A12, heat shock protein, tenascin-C, serum amyloid A, and IL-33. Hyaluronic acid (hyaluronan) (HA) is one of the danger signals. Recent progress has demonstrated that hyaluronan is an important immune regulator in various diseases. In particular, low molecular weight hyaluronan is a ligand for TLRs that induces inflammatory cytokine gene expression. TLRs play an important role in innate immune responses, and in psoriasis, activation of TLR7 and TLR9 via autoimmune plasmacytoid dendritic cell activation releases interferon-α, which further stimulates mDCs to secrete IL-23. Additionally, other TLRs, i.e., TLR2 and TLR4, are also involved in the pathogenesis of psoriasis and psoriatic arthritis [38]. HA is abundant in the psoriatic skin, and hyaluronan fragments signal through TLR4 and TLR2 [39]. In particular, breakdown of high molecular weight HA following injury/damage into low molecular weight HA triggers the release of proinflammatory mediators. CD44, a major cell-surface hyaluronic acid binding protein, is expressed in T-cells. Ligation of CD44 in T-cells and neutrophils induces IL-6 secretion and inflammation [40], and TLR4 is upregulated in the lesional skin of SSc [41]. The ligands of TLR4 are tenascin-C and fibronectin extra-domain A (EDA), and tenascin-C and fibronectin EDA have been found to be elevated in human SSc as well as in a bleomycin-induced murine model [41].

Recently, an imbalance in DAMP release and/or signaling has been found to potentially lead to sustained inflammatory cytokine production by fibroblasts or macrophages, which may be important in the pathophysiology of SSc. pDC and type I IFN production is involved in the innate immunity of the SSc pathogenesis [42,43]. pDCs were detected in the affected skin of SSc patients [44,45], as well as in the bleomycin-induced murine model [44]. Moreover, depletion of pDC reduced fibrosis, immune cell infiltration, and expression of genes and proteins involved in fibrosis [45].

Psoriasis is highly related to metabolic syndrome, and patients often develop obesity, hypertension, diabetes mellitus, hyperlipidemia, and hyperuricemia. It has been remarked that the representative psoriasis comorbidities are cardiovascular diseases and metabolic syndrome, including obesity, hypertension, diabetes mellitus, insulin resistance, hyperlipidemia, and atherosclerotic diseases. Patients with psoriasis are at an increased risk of developing metabolic syndrome, and patients with severe, resistant psoriasis are reported to be significantly more likely to have metabolic syndrome compared with hospital-based controls (odds ratio: 5.92; 95% confidence interval: 2.78-12.8) [46]. Adipose tissues produce several proinflammatory cytokines such as TNF-α, leptin, resistin, and adiponectin, which have been suggested as being responsible for linking obesity and metabolic syndrome. Recent studies showed elevated circulating leptin levels in patients with psoriasis [47]. Leptin is derived from adipocytes and regulates the immune and inflammatory process via proinflammatory cytokine production. Expression of leptin and its receptor was significantly higher in the involved skin of psoriasis [48]. Thus, leptin may play an important role in the induction of psoriasis, especially in patients with obesity. On the other hand, plasma adiponectin levels were found to be decreased in psoriatic patients [49]. Adiponectin has anti-inflammatory effects and regulates insulin sensitivity. A negative correlation was demonstrated between plasma adiponectin levels and both the Psoriasis area and severity index (PASI) score and plasma TNF-α levels in psoriatic patients [49]. Chronic inflammation and persistent release of TNF-α and IL-6 are both produced by adipose tissues and may contribute to the comorbidities of psoriasis and metabolic syndrome. Leptin is a representative adipokine that induces human lung fibroblasts to differentiate from myofibroblasts [50]. Adipokines play various roles in the induction of inflammation, vascular damage, fibroblast proliferation, and collagen production [49].

Dickkopf-related protein-1 (DKK-1) is also a Wnt inhibitor that inhibits osteoblast function [51]. DKK-1 is also induced by TNF signaling [51]. DKK-1 plays an important role in bone remodeling [51]. Recently, the role of DKK-1 in fibrosis was suggested to have a protective role against fibrosis [52]. Expression of DKK-1 in the biopsied scleroderma skin has been reported to be decreased, as compared with normal skin, while circulating levels were found to be normal [53].

Tissue resident memory T-cells (T_RM_) exist in the epidermis of scars only, but to a lesser extent than that of the psoriasis-developed epidermis. It remains unclear whether psoriasis arises only in T_RM_ highly frequent areas or whether psoriatic development simply results in the increment of T_RM_. Psoriasis recurs in previously affected sites, and CD8^+^ T_RM_ enriched in the resolved lesion preferentially produces IL-17 and IL-22 upon restimulation [54,55]. The number of CD8^+^CD103^+^ T_RM_ in the psoriatic epidermis correlates with epidermal thickness, and psoriatic skin-derived CD103^+^T_RM_ produce IFN-γ, IL-17A, and IL-22, suggesting the important roles of T_RM_ in the formation of psoriasis [56]. In addition, epidermis from never-lesional skin from psoriasis patients skews the populations of CD8^+^T_RM_ [57]. Given that scars provide a susceptible environment for psoriasis, epidermal T_RM_ should be elevated in scars and reactivated by additional local or systemic factors. In this scenario, it is possible that unknown factors or antigens activate T_RM_ presumably via T-cell receptors, and the excreted cytokines stimulate keratinocytes to initiate the amplification loop. A very recent report shows that CD8^+^T_RM_ with IL-17A-producing potential are accumulated in psoriatic disease-naïve non-lesional skin, correlated with disease duration [58]. Unfortunately, we could not clarify the frequency of epidermal T_RM_ on non-lesional skin in our patient. Nevertheless, our finding provides another new example of the Köbner phenomenon in which epidermal T_RM_ may be involved. By contrast, studies on T_RM_ in SSc are few in number. CD8+ T-cells are increased in number in the peripheral blood of SSc patients. Recent studies have shown that skin-resident effector memory CD8+CD28- T-cells are increased in the peripheral blood and affected skin of SSc patients [59]. Most CD8+CD28- T-cells in the SSc skin are CD69+CD103- T_RM_, and these T-cells are suggested to induce vascular damage. Furthermore, CD8+CD28-IL-13+ T-cells are profibrotic [59]. By contrast, another study showed a diminishment of CD4+CD103+ T_RM_ in SSc skin [60].

In the cutaneous fibrosis, crosstalk between keratinocytes and fibroblasts may be a clue to understanding the complex pathophysiology. Previously, crosstalk between epidermis and dermis mediated by mast cells was suggested in dermatofibroma, which was proposed a possible local model of cutaneous fibrosis [61]. Recently, contribution of the overlying epidermis in SSc has been highlighted, which plays as a driver or modifier of dermal sclerosis [62]. Keratinocyte-derived IL-1α is suggested to play an important role in stimulating dermal fibroblasts to produce type I collagen.

## 4. Psoriasis and DM

Co-existence of psoriasis and DM is extremely rare, and only several cases have been reported [63,64,65,66,67]. Clinically, keratotic erythemas are frequently observed on the extensor aspect of the elbows and knees in both diseases. A case with DM who developed psoriasis in parallel with exacerbation of interstitial lung disease was previously reported, wherein there was speculation that viral infection caused IFN-α release, leading to the induction of psoriasis [66]. pDC and type I IFN has been suggested in DM [68,69,70], which is shared with the pathogenesis of psoriasis. IFN-inducible proteins such as myxovirus-resistance protein (MxA) and CXC chemokines (CXCL9, CXCL10, and CXCL11) are commonly overexpressed in DM skin and muscle [71]. By contrast, a recent study demonstrated that epidermal expression of MxA differs among groups with different autoantibodies, and is rarely expressed in the finger lesions of patients with anti-aminoacyl transfer RNA synthetase antibody [72]. In addition, serum IL-17 levels are increased in dermatomyositis with a relationship to disease activity [70]. Expression of IL-17 in the cellular infiltrates in both skin lesions may suggest that IL-17 possibly contributes to the development of DM as well as psoriasis.

Serum levels as well as culture supernatant from peripheral blood mononuclear cells were found to be elevated in patients with DM and polymyositis. IL-17 and IL-23 levels were elevated in patients with early disease durations [73]. In a murine model of myositis, IL-23 played an important role in muscle damage [74]. A recent report showed successful use of ustekinumab, an anti-IL-12/23 p40 monoclonal antibody useful for psoriasis therapy, for refractory mechanic’s hand in a patient with antisynthetase syndrome [75].

## 5. Psoriasis and SjS

Type I IFNs, such as IFN-α and IFN-β, drive the inflammatory pathways in the pathogenesis of autoimmune diseases, including rheumatoid arthritis and SjS. Activated CD4+ T-cells, especially IFN-γ-producing Th1 cells and IL-17-producing Th17 cells, contribute to the pathogenesis of SjS [76]. Skin manifestation of SjS includes annular erythema, hypergammaglobulinemic purpura, cryoglobulinemia, as well as various non-specific manifestations such as vitiligo, livedo, xeroderma, localized amyloidosis, and lymphoproliferative diseases [77], and there are several case reports on the association of psoriasis and SjS [78,79,80]. In addition, an increased number of IL-17-positive T-cells was reported in the lesional skin of annular erythema associated with SjS [81]. Therapy targeting IL-23/IL-17 as well as IL-23/IL-12 may be expected for cases involving psoriasis and SjS [82].

## 6. Psoriasis and RA

RA presents various cutaneous manifestations, either specific or nonspecific skin features, which are induced by the activation of inflammatory cells (neutrophils, lymphocytes, macrophages), vasculopathy, vasculitis, acral deformity, drugs, and so on [83]. These include (i) specific findings, (ii) findings due to vascular impairment, (iii) findings due to immune dysfunction, (iv) characteristic neutrophilic conditions, and (v) miscellaneous conditions. It is not uncommon for patients with RA to develop symptoms overlapping those of other connective tissue disorders, such as SSc, SLE, and SjS. Moreover, overlapping cutaneous disorders such as morphea and discoid lupus erythematosus have been reported. Autoimmune bullous dermatoses including bullous pemphigoid, pemphigus vulgaris, pemphigus foliaceus, cicatricial pemphigoid, and linear IgA dermatosis have been reported in RA.

According to a previous study, the highest odd ratio among psoriatic disease in 25,341 psoriasis patients was RA [4], although PsA should be differentiated. Differentiation of PsA and RA is occasionally difficult, although a Th1-dominant cytokine balance has been suggested in both RA and PsA. However, IL-17 blockers are effective for psoriasis and PsA, whereas they did not have favorable effects on RA. By contrast, IL-6 receptor antibody is effective for RA, but its effect on PsA is currently not established.

Mast cell is a rich source of various growth factors and mediators. In RA, mast cells are increased in the synovial tissues. Moreover, mast cells secrete proinflammatory cytokines, angiogenic cytokines, and fibrogenic cytokines. In addition, mast cell-derived proteinases including tryptase and MMPs, such as MMP-2 and MMP-9, are suggested to play a role in the degradation of cartilage.

## 7. Psoriasis and Adult-Onset Still’s Disease

Systemic inflammatory conditions of adult-onset Still’s disease (AOSD) characterized by fever, systemic symptoms (i.e., anemia, arthralgia, liver dysfunction, lymphoadenopathy), and increased levels of acute-phase protein are suggestive of autoinflammatory diseases, in which innate immunity is mainly involved. A predominant shift towards Th1-type cytokines was shown in the peripheral blood and tissues of patients with active AOSD. Serum levels of a number of inflammatory cytokines were significantly higher in patients with active AOSD compared with those in healthy controls. In particular, IL-18 activates Th1-type cytokine response and induces IFN-γ and TNF-α production. IL-18 functions as stimulation of neutrophil migration and activation, enhancement of expression of adhesion molecules, and activation of natural killer cells. IL-18 enhances FasL-mediated cytotoxicity of Th1-type cells [84], and increased apoptosis of peripheral blood lymphocytes is induced in active stage AOSD [85]. IL-18 is also involved in Th17 cell response synergistically with IL-23 [86]. In addition, sustained macrophage activation may result in tissue inflammation; production of ferritin; increased secretion of inflammatory cytokines including IL-1, IL-6, IL-18, IFN-γ, and TNF-α; and reactive hemophagocytic syndrome. Many cell types exemplified by macrophages produce pro-IL-18 that is cleaved by IL-1β-converting enzyme (caspase-1). It has been suggested that IL-18, as well as IL-1, IL-6, and TNF-α, may stimulate ferritin synthesis or inhibit its clearance. IL-18 is suggested to induce IL-1β production. IL-1β is a key mediator of acute inflammation, innate immunity, and adaptive immune response. In addition, recently, IL-1 family proteins such as IL-33 and IL-36 have also been found to be important in autoinflammatory disorders. IL-33 binds to its receptor ST2L, which then activates Myeloid differentiation factor 88 (MyD88) and nuclear factor kB (NF-kB), mediated via IL-1R accessory protein. The trigger of AOSD is supposed to be viral infection, and TLRs are activated, leading to pro-IL-1β and pro-IL-18 synthesis via activation of the NF-kB signaling pathway. Activation of TLR induces enhanced production of IL-1β, IL-6, IL-18, and IFN-α by peripheral blood mononuclear cells [87].

It is well-known that skin rash of AOSD is typically salmon-pink, macular, or maculopapular erythema, which appears in parallel with the onset of fever and disappears in accordance with the decrease in fever; however, recent findings clarify that AOSD presents with various cutaneous manifestations other than typical skin rash [88]. Concurrent psoriasis and AOSD is very rare [89,90], and a case presenting with psoriasiform eruption has been reported [90], but cutaneous manifestation of AOSD does not include psoriasis/psoriasis-like lesions.

Still’s disease was initially reported as a childhood disease. Still’s disease, systemic juvenile idiopathic arthritis (sJIA), and AOSD exist on a spectrum, and it has been generally considered that some cases occurring before age 16 years are sJIA while cases presenting after age 18 years are AOSD [91]. However, there are indeterminate cases in which sJIA is identical to adolescent-onset Still’s disease. The skin rashes of both disorders are similar, and transient, salmon-pink, macular, or urticarial erythema appear on the face, trunk, and extremities, along with fever spikes. Dactylitis is sometimes seen in association with various diseases such as psoriatic arthritis, tuberculosis, injury, gout, and sarcoidosis; however, cases with either sJIA or AOSD presenting with dactylitis have been scarcely reported [92].

## 8. Psoriasis and Behçet’s Disease

Behçet’s disease is a Th1-dominant disease, and recent studies have furthermore shown that Th17-related cytokines are also involved [93]. However, association of Behçet’s disease and psoriasis is rare [94,95]. Musculoskeletal involvement is frequent in Behçet’s disease, and enthesopathy is also observed in Behçet’s disease. Recently, BD is proposed to be included in “MHC-I-opathy” with prevalence of HLA-B 51, in which CD8+ T-cells activate neutrophils and drive inflammation [96,97]. Apremilast blocks phosphodiesterase-4, which regulates immune and inflammatory processes through modification of the levels of intracellular cyclic adenosine monophosphate, protein kinase A, and various forms of inflammatory cytokine production. Apremilast is used for both psoriasis and Behçet’s disease.

## 9. Psoriasis and Granulomatous Diseases

Non-infectious granulomatous conditions such as granuloma annulare and sarcoidosis are rarely associated with psoriasis [98]. Th1 type cytokines are favored in the initial phase of sarcoidosis. In particular, TNF-α is important in the formation of sarcoidal granuloma [99]. A shared TNF-α-mediated pathogenesis between psoriasis and sarcoidosis may exist. TNF-α activates Th17 cells to lead IL-17 production, and the IL-17 inflammatory pathway has been suggested to be important in psoriasis. Moreover, recent studies have suggested an important role of IL-17 in sarcoidosis, and enhanced expression of IL-17A+IFN-γ+ and IL-17A+IL-4+ memory T-cells was shown in sarcoidal lungs [100]. Thus, Th17 profile has been implicated to play a role in sarcoidosis, possibly by inducing granuloma formation via suppression of regulatory T-cells [100]. Another study showed upregulation of IL-23 and IL-21 in the lesions of cutaneous sarcoidosis [101]. In addition, pso p27 is a protein detected in mast cells in psoriatic lesions and extractable from psoriatic scales. Pso p27 is abundantly expressed in psoriatic lesional skin, and also expression of pso p27 is increased in the lungs of pulmonary sarcoidosis [102]. Further study is needed to determine the role of pso p27 in sarcoidosis.

Granuloma annulare has been considered to be driven by a Th1-mediated process via upregulation of TNF-α. Moreover, MMPs such as MMP-2 and MMP-9 are important to degrade the extracellular matrix, leading to necrobiosis in the center of granuloma annulare. Recent studies have shown Th2 (i.e., IL-4 and IL-31) and Janus kinase pathways are involved in the pathogenesis of granuloma annulare [103]. Alternatively, IL-17 was abundantly detected in psoriasis, which may induce granuloma formation by suppressing regulatory T-cells.

## 10. Conclusions

Several external triggers have been proposed not only in psoriasis but also in connective tissue disorders (Table 1). They are termed the Köbner phenomenon, internal Köbner phenomenon, photo-Köbner phenomenon, deep Köbner phenomenon, and so on. Infection and drugs are also important precipitating factors. Cases of association with other disorders or drug-induced psoriasis provide a good opportunity for approaching the pathogenesis of psoriasis as well as connective tissue diseases. Further studies are necessary to gain deeper insights into the pathogenesis of, and eventually lead to new therapies for, psoriasis and connective tissue diseases.

## Figures and Tables

**Table 1:** Possible precipitating external factors for the induction of skin lesions of psoriasis and connective tissue diseases.

	External Triggers for Induction of Skin Lesions
Psoriasis	Köbner (physical stress, vaccination, minor trauma, etc.) Drug Infection Microorganism
SLE	Photo-Köbner (ultraviolet) Drug
SSc	Coldness Köbner for calcified nodule
Dermatomyositis	Köbner for Gottron’s sign
RA	Köbner for rheumatoid nodule
SjS	Unknown for annular erythema
